# An Adult With Dyke–Davidoff–Masson Syndrome: A Case Report

**DOI:** 10.7759/cureus.23315

**Published:** 2022-03-19

**Authors:** Ali Al-Smair, Sufian Abdel Hafez, Ahmad Saadeh, Ahmad Al-Ali

**Affiliations:** 1 Department of Radiology, Medray International Radiology Center, Amman, JOR; 2 Faculty of Medicine, The University of Jordan, Amman, JOR; 3 Department of Radiology, Jordan Ministry of Health, Amman, JOR

**Keywords:** dyke–davidoff–masson syndrome, seizure, cerebral hemiatrophy, adult presentation, case report

## Abstract

Dyke-Davidoff-Masson syndrome (DDMS) is a rare disease affecting the brain with almost 100 cases previously reported, with only 21 cases among adults. Due to the intricacy of clinical manifestations and radiological findings, it is difficult to reach the diagnosis. It usually includes atrophy of the cerebral hemisphere, dilation of the lateral ventricle, hypertrophy of skull bones, and hyperpneumatization of air sinuses. Herein, we present a case of a 55-year-old female patient who presented with a new-onset seizure. This case emphasizes the importance of considering DDMS in the differential diagnosis of adult-onset seizures, especially in patients with a previous history of brain insult, and demonstrates the possibility of developing this condition despite the lack of childhood symptoms. To our knowledge, this is the first case reported in Jordan.

## Introduction

Dyke-Davidoff-Masson syndrome (DDMS) is a rare neurological condition with the main characteristic of cerebral hemiatrophy on brain imaging [1]. Since 1933 when it was described for the first time, almost 100 cases have been reported [2], while only 21 cases have been reported in adults [1]. It is described by the presence of cerebral hemiatrophy, dilation of the lateral ventricle on the ipsilateral side, compensatory hypertrophy of skull bones, and enlargement of the frontal and ethmoidal sinuses [3]. Patients usually present with seizures, hemiplegia or hemiparesis, facial asymmetry, and cognitive or psychiatric symptoms [4]. Herein, we present a case of a 55-year-old female patient who presented with a new-onset seizure, with a remote history of stroke 32 years before this presentation.

## Case presentation

A 55-year-old female patient presented to the emergency department following a generalized tonic-clonic seizure for the first time. After ensuring the stability of the patient, a thorough history was taken from her and her family. She had an unremarkable childhood with normal developmental milestones, until the age of 23 when she had a stroke. Workup for vasculitis, thrombophilias, and vascular embolisms was unremarkable at the time of the stroke, and no clear cause was established. On physical examination, she had facial asymmetry and hemiparesis. Muscle strength on the right upper limb and lower limb was 3/5, while the left upper and lower limbs had a score of 5/5. Upper and lower limbs’ sensation and proprioception were normal. Additionally, she scored 27 out of 30 on the Mini-Mental State Examination (MMSE). Laboratory tests and a septic workup were ordered in which normal results were found.

**Figure 1:**
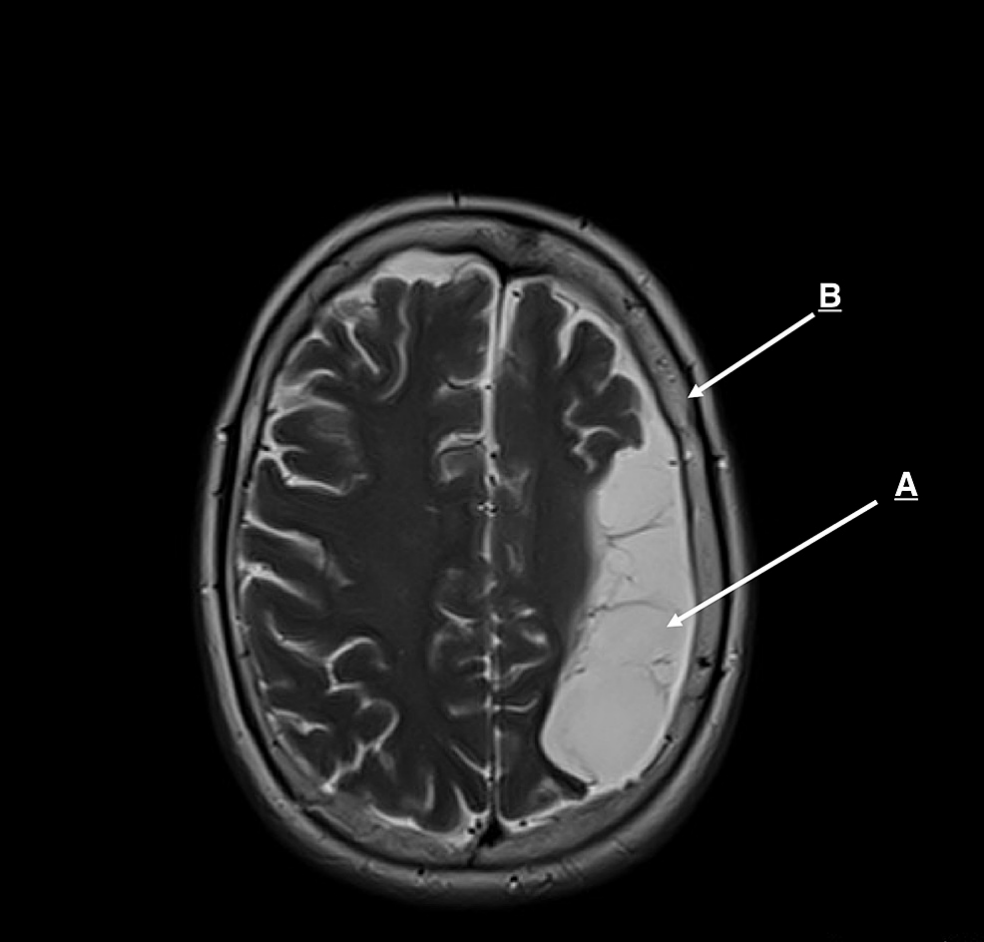
A: Encephalomalacia of the left frontoparietal lobes corresponding to a previous infarction of the left MCA territory. B: Associated skull vault thickening.

On magnetic resonance image (MRI), axial T2 image showed extensive encephalomalacia changes in the left frontotemporoparietal lobes, indicating left cerebral hemiatrophy corresponding to a previous infarction of the left middle cerebral artery (MCA) territory, and associated skull vault thickening (Figure 1).

**Figure 2:**
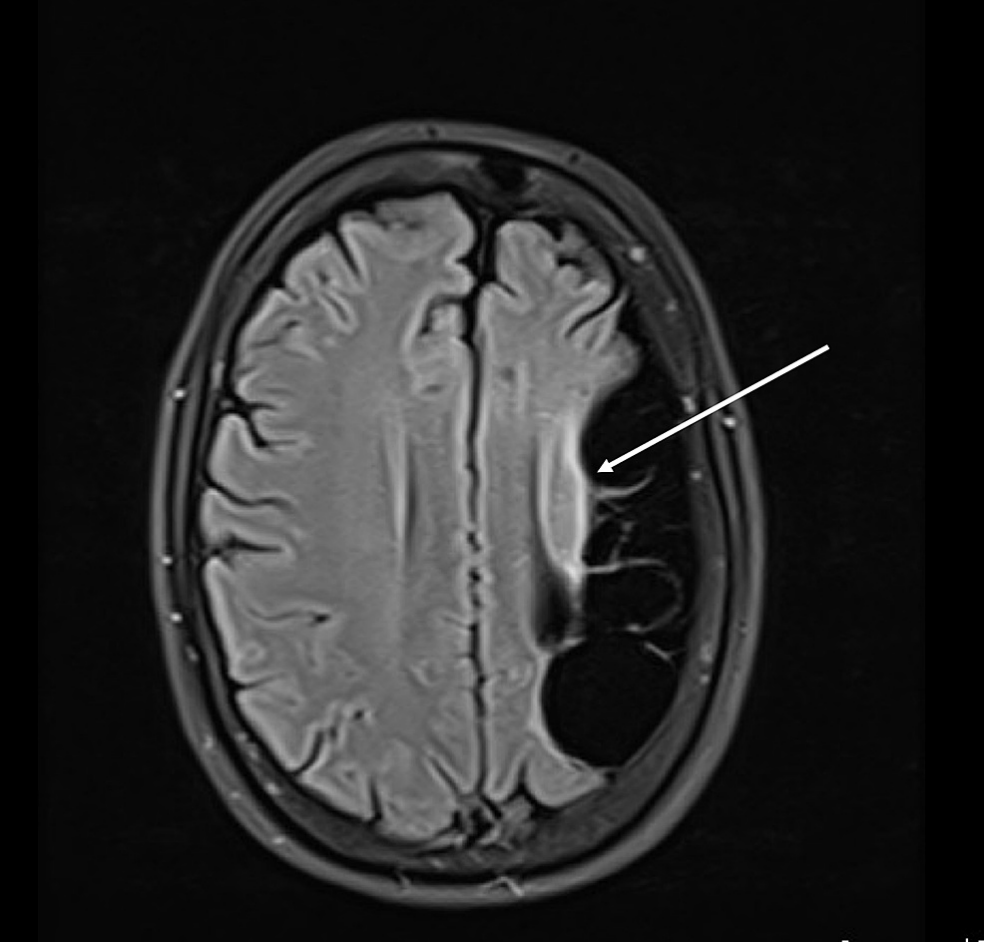
Mild gliosis associated with encephalomalacia (arrow).

On the axial flair image, gliosis of the frontotemporal lobe as a result of the previous insult was evident (Figure 2).

**Figure 3:**
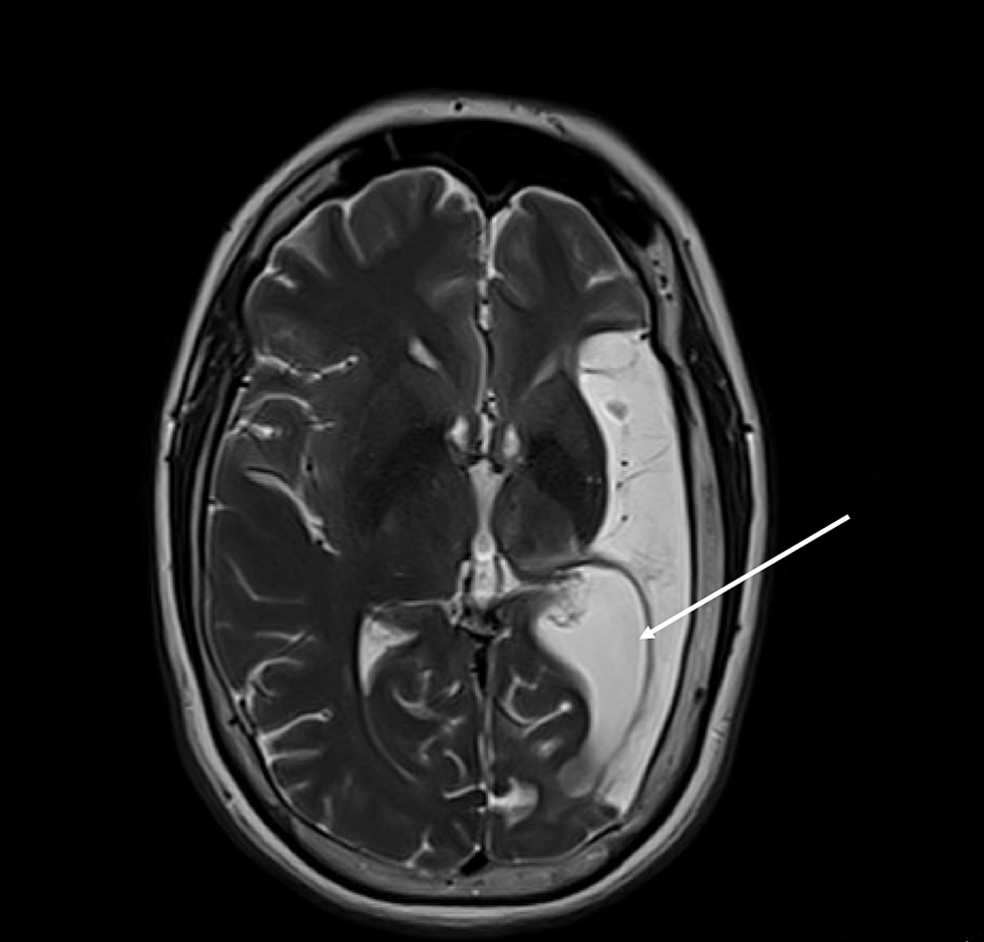
Ex vacuo dilatation of the occipital horn of the lateral ventricle (arrow).

An ex vacuo dilatation of the left occipital horn of the lateral ventricle was also found on the axial T2 image (Figure 3).

**Figure 4:**
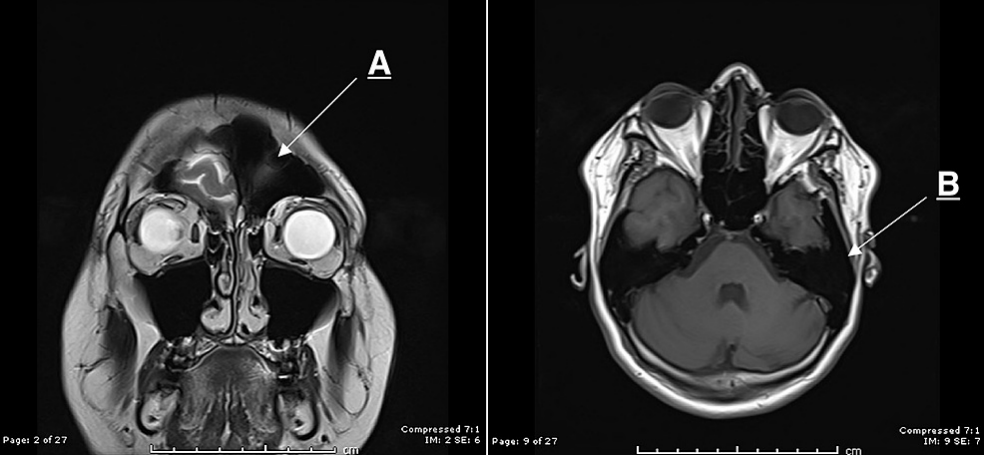
A: Hypertrophied left frontal sinus. B: Hypertrophied left mastoid air sinus.

Enlargement of the left frontal sinus and left mastoid air cells were found as well (Figure 4).

**Figure 5:**
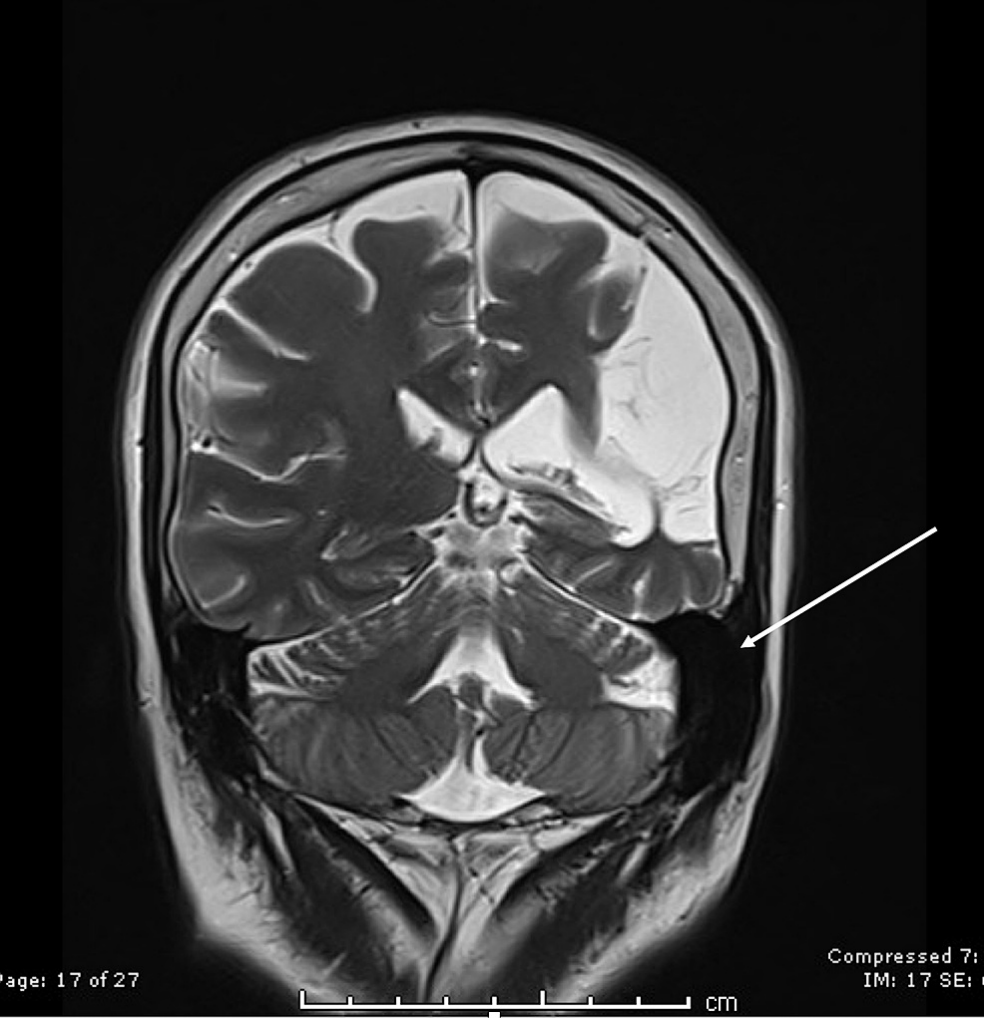
Elevation of the left petrous bone (arrow).

There is also an elevation of the petrous ridge (Figure 5).

All of the previous findings are classical characteristics of DDMS, which along with the clinical presentation were enough to make the diagnosis. The patient was started on valproic acid to prevent seizure recurrence.

## Discussion

Dyke-Davidoff-Masson syndrome (DDMS) is a rare neurological condition named after the three physicians who first described it in 1933 with nine patients presenting with hemiparesis, facial asymmetry, seizures, intellectual disability, and skull radiography findings of pneumatoencephalographic changes [5]. The pathogenesis of DDMS is still not clear, but previous studies have attributed the condition to a brain injury either in utero, which presents in infancy (congenital subtype), or early childhood (acquired subtype) due to a variety of possible reasons such as trauma, ischemia, hemorrhage, and infection [2]. Although DDMS is widely regarded as a pediatric condition, previous cases in the adult population have been described, with only 21 cases reported in a recent review [1]. Our patient presented with a seizure, hemiparesis, and facial asymmetry and had clear radiological evidence of DDMS at the age of 55.

DDMS presents clinically with a variety of possible features that may differ in severity depending on the degree of injury. Seizures (both focal and generalized), facial asymmetry, contralateral hemiparesis, and intellectual disability are some of the more prominent features [6]. Associated neuropsychiatric symptoms and psychiatric disorders have been described as well [7]. The previous history must be carefully explored for any symptoms, and special attention should be given to the developmental pediatric history since this is the typical time of presentation. A proper neurological and cognitive examination is essential in assessing this condition as well. Our patient presented with a generalized tonic-clonic seizure and examination findings of right-sided weakness and facial asymmetry, which are consistent with the condition, although her mental examination was unremarkable.

In addition to the clinical presentation, radiological findings of CT scans and MRI play a core role in establishing the diagnosis and assessing the severity of the condition. These findings vary from one patient to another and typically include atrophy of the cerebral hemisphere at the side of the lesion, along with lateral ventricular dilation and prominent sulci. Other prominent changes include compensatory hypertrophy of skull bones and hyperpneumatization of air sinuses such as the frontal sinus [8]. It has been suggested that a compensatory calvarial thickening occurs when the brain injury has happened before three years of age or during the intrauterine period [5]. Our patient had hypoplasia of the left cerebral hemisphere with ventricular dilation, skull thickening, and left frontal and mastoid sinus enlargement consistent with the compensatory changes seen in DDMS. Her previous history of stroke could be the triggering factor in developing this condition, although no previous images are available for comparison.

The management approach of DDMS is centered on symptomatic improvement. Anticonvulsant medications for seizure control, physiotherapy for neurological improvement, and speech therapy are some of the modalities used, depending on patients’ needs. In cases of hemiplegia with intractable disabling seizures, hemispherectomy is indicated with a reported 85% success rate [9]. Patients with recurrent and prolonged seizures and those in whom hemiparesis commences before two years of age have a worse prognosis [10]. Our patient was started on valproic acid to prevent recurrent seizures in a similar approach to that taken by other reported cases since there are no clear guidelines for the management of seizures in these patients [6].

## Conclusions

Dyke-Davidoff-Masson syndrome (DDMS) is a neurological condition that presents with a constellation of symptoms with various severities that can include seizures, hemiparesis, and intellectual disability with characteristic cerebral hemiatrophy, skull hypertrophy, and sinus hyperpneumatization. Despite being regarded as a childhood condition, adult presentation is possible even without a suggestive pediatric history. Symptomatic management is the mainstay of treatment, although surgical intervention is indicated as a last resort in certain cases.
